# Corneal edema associated with degenerating Soemmering ring cataract: Clinical-pathologic correlation

**DOI:** 10.1016/j.ajoc.2022.101738

**Published:** 2022-11-11

**Authors:** Jordan P. Safran, Nathan Nataneli, Jayesh Vazirani, Ralph C. Eagle Jr, Tatyana Milman

**Affiliations:** a Department of Pathology, Wills Eye Hospital, Thomas Jefferson University, Philadelphia, PA, USA; b BronxCare Health System, Bronx, NY, USA; c Excel Eye Care, Ahmedabad, India; d Department of Pathology, Anatomy, and Cell Biology, Sidney Kimmel Medical College of Thomas Jefferson University, Philadelphia, PA, USA

**Keywords:** Soemmering ring, Calcific Soemmering ring, Calcific deposits cornea, Dispersing Soemmering ring, Degenerating Soemmering ring, Cataract surgery complication

## Abstract

**Purpose:**

To report three patients with an uncommon delayed complication of cataract extraction: corneal edema following dispersion of calcific lens particles from a degenerating Soemmering ring cataract.

**Observations:**

We report three patients, 75–92 years old, presenting with corneal edema and dispersed, degenerated calcific lens material in the anterior chamber and vitreous 20–30 years after cataract surgery. In all patients, calcific particles studded the posterior surface of the cornea in a gravity-dependent distribution without apparent inflammation and were associated with localized corneal edema. In one patient, calcific particles were also associated with secondary open angle glaucoma. Deposits originated from the calcified Soemmering ring cataract. Histopathological examination demonstrated extracellular calcific deposits compatible with cataractous lens material on the posterior surface of stripped Descemet membrane of two patients. The deposits were associated with prominent localized loss of corneal endothelium and were not associated with inflammation. Morphologically similar acellular material was identified in the biopsied aqueous and vitreous fluid of one patient. Management included endothelial keratoplasty, anterior chamber lavage, pars plana vitrectomy, aspiration/removal of a portion of Soemmering ring cataract without intraocular lens implant explantation, and the removal of the entire capsular bag/implant complex. Cornea cleared and visual acuity improved in both patients who underwent endothelial keratoplasty. Persistent elevated intraocular pressure led to visual deterioration in one patient with secondary glaucoma.

**Conclusions and Importance:**

Dispersion of calcific Soemmering ring cataract can occur decades following cataract surgery leading to corneal edema, secondary glaucoma, and vitreous opacities. Timely recognition of this phenomenon may prevent ocular morbidity, including corneal edema and glaucoma.

## 1 Introduction

Soemmering ring cataract is an annular-shaped structure composed of residual cataractous lens material sequestered within the equatorial portion of the lens capsular bag following cataract extraction or non-surgical trauma.^1^ The residual lens cortex and the continued proliferation of lens epithelial cells contribute to Soemmering ring cataract formation. In most cases, Soemmering ring cataract remains hidden innocuously behind the iris. Less frequently, Soemmering ring cataract can directly block the visual field, become dislocated, or become liquefied leading to visual disturbances, glaucoma, and uveitis.^1^, ^2^, ^3^

Herein, we describe three patients with remote history of cataract extraction, who developed dispersion of calcific lens particles from the fragmenting, degenerating Soemmering ring cataract, leading to corneal edema.

This study was approved by the Wills Eye Hospital Institutional Review Board.

## 2 Findings

### 2.1 Case 1

**Fig. 1:**
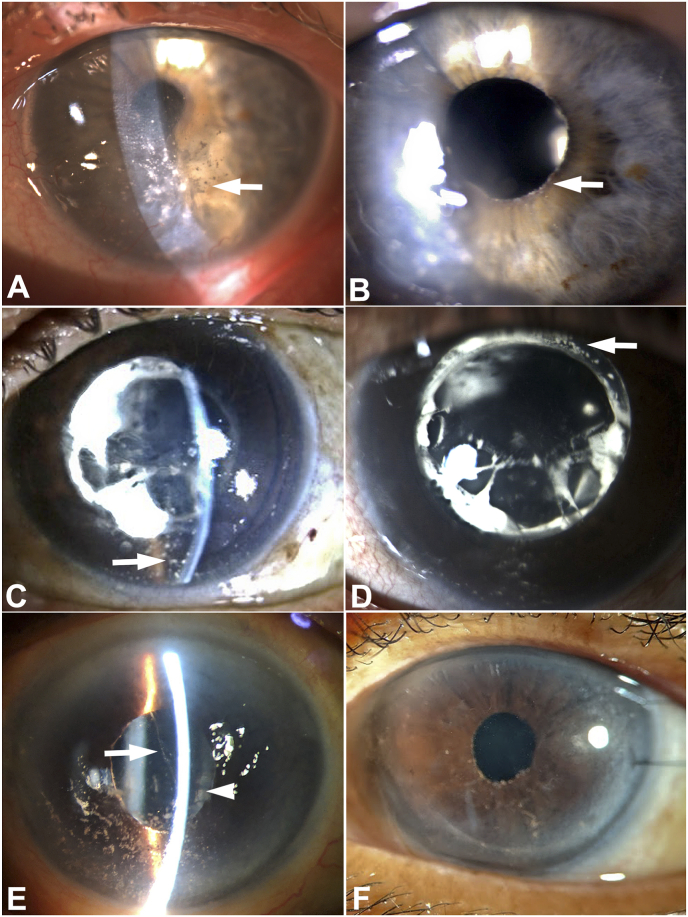
**Degenerating Soemmering ring cataract: clinical presentation**. Patient 1: Slit lamp photograph demonstrates variably sized white chalk-like and powdery talc-like deposits on the posterior corneal surface (arrow) in gravity-dependent distribution, associated with localized corneal edema (**A**). Powdery deposits also are present at the pupillary margin (arrow) (**B**). Patient 2: White granular deposits in gravity-dependent distribution on the posterior cornea (arrow), associated with edema (**C**). Calcific white Soemmering ring cataract is present adjacent to the posterior chamber intraocular lens implant (**D**). Patient 3: White powdery deposits on the posterior cornea in gravity-dependent distribution and in the anterior chamber (arrow). Soemmering ring cataract is present (arrowhead) (**E**). Clear cornea following endothelial keratoplasty (**F**).

A 92-year-old woman was referred for evaluation of persistent corneal edema in the left eye. Her past medical history was significant for hypertension, chronic obstructive pulmonary disease, and gout managed with fluvastatin, olmesartan medoxomil, albuterol, furosemide, aspirin, and allopurinol. Past ocular history was notable for uncomplicated bilateral extracapsular cataract surgery with posterior chamber intraocular lens (PCIOL) placement (implant model unknown) 20 years prior, followed by posterior yttrium-aluminum-garnet (YAG) capsulotomy over ten years ago. There was no history of trauma, uveitis, or elevated intraocular pressure. She was previously treated empirically with methylprednisone drops for corneal edema by the referring provider; but this therapy did not improve the condition and was discontinued. At the time of referral, visual acuity with correction was 20/40 in the right eye and finger counting in the left eye. There was a relative left afferent pupillary defect. Intraocular pressure (IOP) was normal: 11 mm Hg in the right eye and 12 mm Hg in the left eye. Examination of the right eye was notable for a clear cornea with trace guttae, pseudophakia, and otherwise unremarkable anterior and posterior segments. Examination of the left eye revealed a white powdery precipitate on the corneal endothelium centrally and inferiorly, associated with the edema of the overlying central and inferior cornea (Fig. 1). Similar deposits were identified in the inferior anterior chamber angle and on the pupillary border of the poorly dilating iris (Fig. 1). A PCIOL was present in the lens capsular bag. Funduscopic exam and ocular coherence tomography (OCT) of the optic nerve and macula of the left eye were limited by corneal edema.

**Fig. 2:**
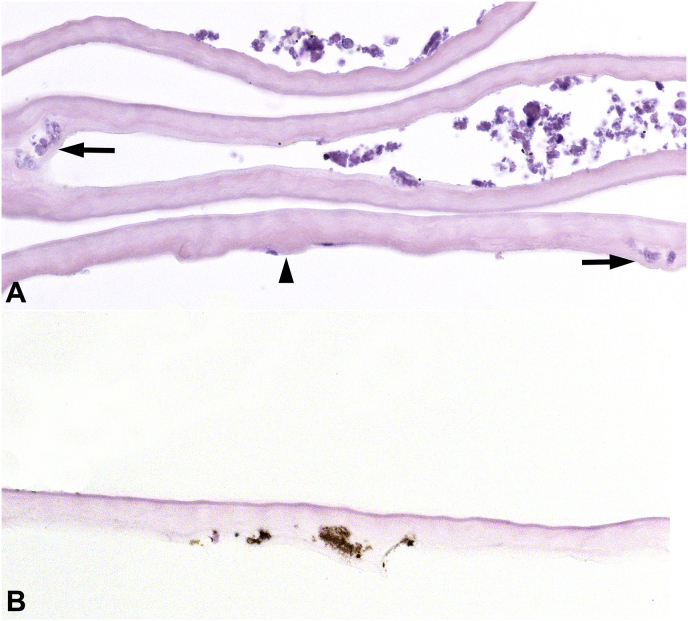
Degenerating Soemmering ring cataract: histopathologic findings in Patient 1. Thickened Descemet membrane with guttae (arrowhead) and severe endothelial cell loss. Numerous basophilic granular deposits are present, focally incorporated into posterior collagenous layer (arrows) (**A**). The deposits stain brown with Von Kossa stain for calcium (**B**). [stains: hematoxylin-eosin (A), von Kossa (B); x400]. (For interpretation of the references to colour in this figure legend, the reader is referred to the Web version of this article.)

The operative treatment for this patient included a superficial punctate keratotomy, Descemet membrane stripping automated endothelial keratoplasty (DSAEK), and 3-port pars planar vitrectomy (PPV). Intraoperatively, white talc-like particles were noted to exude from the space between the anterior lens capsule and the lens optic into the anterior chamber. Identical white chalk-like deposits were identified on the posterior surface of stripped Descemet membrane. Similar white talc-like particles were apparent in the vitreous, extruding from the space between YAG capsulotomy and the optic. The Vitrector was used to remove a significant amount of degenerating, crumbling, calcific Soemmering ring cataract via the openings between the anterior capsulotomy and YAG capsulotomy and the lens optic. Minimal residual hard, cohesive, equatorial lens material was left in the lens capsular bag. Histopathologic evaluation demonstrated a thickened Descemet membrane with few central guttae, compatible with Fuchs corneal endothelial dystrophy. Numerous granular and globular, variably sized, irregular, focally eosinophilic and focally basophilic, nonbirefringent extracellular deposits, morphologically compatible with cataractous lens material, were present on the posterior surface of Descemet membrane and focally in the posterior collagenous layer, compatible with chronicity of the process (Fig. 2). The Von Kossa stain confirmed the focal presence of calcium within the deposits (Fig. 2). The deposits were associated with prominent localized loss of corneal endothelium (Fig. 2). No inflammatory cells were identified. An immunohistochemical stain for cytokeratin 8 (CAM5.2) highlighted cellular corneal endothelium lining Descemet membrane distal to deposits and the absence of corneal endothelium adjacent to the deposits. The CD68 immunohistochemical stain confirmed the absence of macrophages. Morphologically similar acellular calcific lens material was identified in the biopsied aqueous and vitreous fluid containing the aspirated calcific Soemmering ring cataract.

Post-operatively, at 6 months follow-up, the cornea remained clear with a well-positioned graft. The visual acuity improved to 20/150 and the IOP remained normal. As the cornea cleared, funduscopic exam revealed a pale optic nerve without appreciable cupping suggestive of ischemic optic neuropathy, for which the patient will undergo further evaluation.

### 2.2 Case 2

A 75-year-old woman presented with progressive visual loss from 20/70 to 20/200 in the left eye over the course of one year, associated with whitish deposits on the corneal endothelium, poorly controlled IOP, and corneal edema. Past medical history included type II diabetes mellitus, hypertension, coronary artery disease, asthma, and hyperlipidemia. Past ocular history was notable for bilateral extracapsular cataract extraction with 3-piece polymethyl methacrylate PCIOL placement 30 years previously, bilateral panretinal photocoagulation 8 years previously, and bilateral primary open angle glaucoma diagnosed 8 years previously, managed with brimonidine/timolol, bimatoprost, and dorzolamide. Exam of the left eye was notable for elevated IOP, open anterior chamber angle, a PCIOL within the lens capsular bag, YAG capsulotomy, and a Soemmering ring cataract with prominent white calcification. The calcific particles appeared to be focally dispersed within the lens capsular bag and were believed to be the source of similar-appearing calcific debris on the corneal endothelium and in the anterior chamber angle (Fig. 1). Over the course of the following several months, there was an increase in white deposits on the corneal endothelium, associated with worsening of corneal edema, continued poorly controlled intraocular pressure in the left eye, and further deterioration of vision to finger counting. There was no observable inflammation in the eye at any point; the intraocular pressure was well-controlled medically in the right eye. It was proposed that the corneal edema and elevated IOP in the left eye resulted from the calcific debris depositing on the corneal endothelium and in the trabecular meshwork originating from the Soemmering ring cataract.

The treatment plan was to perform PPV with removal of PCIOL/Soemmering ring cataract followed by DSAEK and scleral-fixated IOL. Intraoperatively, the view was obscured by corneal edema; however, there was abundant talc-like precipitate extending from the Soemmering ring cataract through space between the PCIOL optic and a large anterior capsulotomy, on the corneal endothelium, and in the anterior chamber angle, which was anatomically open. There was also a space noted between the posterior capsulotomy and the PCIOL. During Soemmering ring cataract removal, several pieces of calcium were dropped onto the retina, which could not be lifted off the macula with perfluorocarbon or removed with a vitrector or fragmatome. These fragments were manually brought up to the anterior chamber. The anterior chamber angle was flushed out with the balanced salt solution and some of the superficial white powdery material from the anterior chamber angle and from the posterior corneal surface was removed with irrigation/aspiration. However, most of the talc-like material from the posterior corneal surface could not be removed. Pathologic evaluation of explanted Soemmering ring cataract documented grossly calcific crystalline lens material. Microscopic evaluation of Soemmering ring cataract was not performed.

Postoperatively, the patient continued to have persistently elevated intraocular pressure up to 30 mm Hg on maximal medical therapy with hand motions visual acuity. A tube shunt was offered, but the patient declined further surgery.

### 2.3 Case 3

An 81-year-old woman who presented with blurred vision in the left eye for 4–5 months. Her past medical history included epilepsy treated with carbamazepine and osteoporosis for which she was taking vitamin D supplements. Past ocular history was notable for retinal detachment surgery in the left eye 30 years prior, bilateral cataract extraction with PCIOL (model unknown) placement 22 years ago, and age-related macular degeneration managed by intraocular injections in the right eye. She was diagnosed with “vitritis” in the left eye 1 year ago, treated initially with oral and topical steroids, followed by PPV with steroid injection at the time of surgery. Vitreous material was not submitted for cytologic and microbiologic evaluation. On examination, visual acuity was finger counting in both eyes. IOP was 10 mm Hg in the right eye and 12 mm Hg in the left eye. The right cornea was clear. The left cornea exhibited central and inferior stromal edema with Descemet membrane folds. White particulate deposits were observed on the central and inferior corneal endothelium and on the iris. A strand of white particles was also present anterior to the PCIOL and calcific Soemmering ring cataract that contained similar-appearing deposits (Fig. 1). The left pupil was dilated poorly. Retinal findings in both eyes included drusen, optic discs with 0.3 cup-to-disc ratio, and healthy neuroretinal rims. A macular scar was present in the right eye. There was no evidence of active inflammation in either eye.

Work-up for systemic etiologies of calcium deposits, including complete blood count with peripheral smear, serum electrolytes, and serum parathyroid levels, was within normal limits. The patient underwent left DSAEK, anterior chamber lavage, release of peripheral anterior synechiae from the 6 o'clock position, and debridement of calcific deposits from the surface of the iris, PCIOL, and Soemmering ring cataract, leaving the lens capsular bag/PCIOL complex intact. Intraoperatively, the particles were noted to be present in continuity over the anterior surface of the IOL, extending from the calcific Soemmering ring cataract via the space between the IOL and the lens capsule. Histopathology of stripped Descemet membrane demonstrated focal calcific deposits on its posterior surface, associated with iris melanin pigment granules and loss of corneal endothelium. No inflammatory cells or old keratic precipitates were noted.

Postoperatively, corneal edema in the left eye resolved (Fig. 1). At two years post-op follow-up, visual acuity in the eye improved to 20/40 and the graft remained stable.

## 3 Discussion

Soemmering ring cataract is not an uncommon sequela of a planned cataract extraction with PCIOL placement. Soemmering ring cataract is typically composed of residual equatorial cortical material with various stages of degeneration, variable proportion of swollen lens epithelial cells (bladder cells/Elschnig pearls), and fibrous metaplasia of lens epithelium, enclosed within the anterior, equatorial, and posterior portions of the lens capsule.^4^

Dystrophic calcification of the cataractous crystalline lens is a well-recognized, complex phenomenon that occurs in areas of cortical degeneration. The dense, hard, globular calcific deposits in cataractous crystalline lens are predominantly composed of calcium phosphate (calcium hydroxyapatite).^5^, ^6^, ^7^, ^8^ Pathogenesis of crystalline lens calcification is not well-understood. Alteration in the intraocular fluid and intra-lenticular calcium homeostasis has long been recognized to be of key importance in cataract development. Calcium levels in the lens are regulated by multiple complex mechanisms, including sodium/calcium exchanger, voltage and receptor-operated calcium channels, calcium ATPases, and various signaling pathways, including receptor tyrosine kinase-mediated signaling pathways.^9^ Increased calcium concentration in crystalline lens leads to early mineral deposition, or nucleation, with initial formation of amorphous calcium phosphate and subsequent conversion to crystalline hydroxyapatite.^7^ These regulatory mechanisms and the calcium levels in the aqueous fluid can be affected by exposure to the ultraviolet light and ionizing radiation,^10^^,^^11^ prolonged hypoglycemia, hyperglycemia, hypoxia, and inflammation.^5^ Our patients had complex medical histories, notable for gout and chronic obstructive pulmonary disease in patient 1 and diabetes mellitus and coronary artery disease in patient 2, which may have predisposed to calcification through various above-mentioned mechanisms. Posterior lens capsule calcification can also occur in a setting of systemic disturbances in calcium and phosphate metabolism, such as hyperparathyroidism.^12^ Patient 3 was noted to receive Vitamin D supplementation. Hypervitaminosis D can contribute to hypercalcemia; however, at the time of her work-up, no systemic alterations of calcium levels were noted. Review of our patients’ known medical histories did not disclose any other systemic medications that could have been associated with calcium and phosphate metabolism disturbances or ocular calcification. None of our patients, to our knowledge, were exposed to topical or intraocular agents associated with crystalline lens calcification. Hypothetically, calcification of the intraocular lens implant itself can serve as a nidus for Soemmering ring cataract calcification. Calcification of the intraocular lenses has been associated with certain implant models, such as hydrophilic acrylic intraocular lenses, asteroid hyalosis, diabetes mellitus, inflammation, and silicone oil installation and other intraocular surgeries.^13^ None of our patients had grossly apparent opacification/calcification of the implant itself; thus, this mechanism seems to be unlikely.

Retained lens material following cataract surgery can cause ocular morbidity by several mechanisms. Phacoantigenic uveitis is characterized by a zonal granulomatous reaction to lens particles.^14^^,^^15^ In a phacolytic response degenerated lens protein is phagocytosed by macrophages, which occlude trabecular meshwork leading to secondary open angle glaucoma.^16^, ^17^, ^18^ Retained lens fragments can cause mechanical damage to corneal endothelium and mechanical obstruction of the trabecular outflow.^19^ Our three patients all demonstrate a distinct, to our knowledge previously unreported, mechanism of delayed post-operative complication of cataract extraction – the dispersion of minute, talc-like calcific particles from fragmenting or degenerating Soemmering ring cataract. In all patients the particles were visualized in the lens capsular bag of degenerating Soemmering ring cataract. In patient 1, histopathologic correlation of the aspirated Soemmering ring cataract with the deposits on Descemet membrane demonstrated identical material, supporting the hypothesis that degenerated calcific cataractous lens material escaped from the space between the lens capsular bag and the optic. While the exact mechanism of this dispersion of calcific cataractous lens material is speculative, we hypothesize that in our patients the extreme cortical liquefaction accompanied by the loss of protein volume, combined with calcification of residual crystalline material led to formation of a potential space between the anterior capsulotomy and the optic, observed intraoperatively in all 3 patients, allowing the talc-like particles to escape into the anterior chamber. Analogously, YAG capsulotomy may have allowed the escape of these particles into the anterior vitreous of patient 1, mimicking asteroid hyalosis. An alternative hypothesis, which is less likely in light of intraoperative observations in our patients, is escape of degenerated lens cortical material through intact lens capsule with subsequent calcification in the anterior chamber and vitreous. Finally, it is possible that deposits on the cornea are not related to Soemmering ring cataract. In the absence of other etiologies of corneal deposits and given their similarity to the process in the Soemmering ring cataract, this last hypothesis seems to be unlikely.

In all three patients, calcific particles studded the posterior surface of the cornea in a gravity-dependent distribution without apparent inflammation and were associated with loss of corneal endothelium, leading to secondary corneal edema. In patient 2, calcific particles were also associated with secondary open angle glaucoma, presumably from mechanical obstruction of trabecular meshwork. Neither patient 1 nor patient 2 had documented inflammation at any point of the clinical course, suggesting that mechanical damage to corneal endothelium and mechanical occlusion/damage to the anterior chamber angle were the leading pathologic mechanisms for corneal edema and open angle glaucoma, respectively. Reportedly, patient 3 had a prior history of vitritis and inferior peripheral anterior synechiae, suggesting that an inflammatory process could have complicated the clinical course. However, histopathology of explanted Descemet membrane disclosed no active inflammation or evidence of remote inflammation.

The differential diagnosis of intraocular calcific deposits includes asteroid hyalosis. Asteroid hyalosis typically is observed in the vitreous, where asteroid bodies are associated with vitreous collagen fibrils. Rarely, asteroid hyalosis can migrate into the anterior chamber of a pseudophakic eye, where these deposits are either associated with prolapsed vitreous gel or can occur in isolation as linear yellow-white multilobular densities in the inferior anterior chamber.^20^ Similar to crystalline lens calcification, asteroid bodies are composed of calcium phosphate (hydroxyapatite).^21^ However, unlike the minute, talc-like, nonbirefringent or weakly birefringent deposits dispersed from Soemmering ring cataract, asteroid hyalosis is composed of larger globules, which demonstrate characteristic maltese cross birefringence with polarization microscopy.^22^

Management of calcific deposits from Soemmering ring cataract included DSAEK for corneal edema in two patients, aspiration/removal of calcific lens fragments without PCIOL explantation in two patients, and the removal of the entire capsular bag/PCIOL complex in one patient. Removal of calcific Soemmering ring cataract can be complicated by dispersion of sharp, calcific particles posteriorly, as exemplified by the intraoperative course of patient 2. This observation, in part, influenced the decision to debride loose calcific material as opposed to removal of the entire Soemmering ring cataract in patients 1 and 3. It is noteworthy that vision improved with resolution of corneal edema and stable DSAEK grafts in both of these patients. However, as their follow-up is limited, 6 months and 2 years, respectively, the long-term outcomes of this management approach for degenerating Soemmering ring cataract are yet to be elucidated. Notably, calcific deposits were integrated into Descemet membrane on histopathologic evaluation (patient 1). Similarly, calcific deposits could not be removed by aspiration alone from Descemet membrane and the anterior chamber angle in patient 2, leading to persistent corneal edema and elevated intraocular pressure. This highlights the importance of timely diagnosis of degenerating Soemmering ring cataract.

Our study is limited by the retrospective design and incomplete information regarding the original cataract surgery and the type of implant placed, precluding a more detailed assessment of the potential risk factors for the development of this post-operative complication.

## 4 Conclusions

In summary, we describe the unusual delayed complication of cataract extraction, the degenerating calcific Soemmering ring cataract. Timely recognition of this phenomenon may prevent ocular morbidity, including corneal edema and glaucoma.

## Patient consent

Consent to publish the case series was obtained from all patients.

## Funding

This study was supported by the Eye Pathology Education and Research LLC, Jenkintown, PA.

## Authorship

All authors attest that they meet the current ICMJE criteria for authorship.

## Research ethics

IRB approval was obtained (required for studies and series of 3 or more cases).

Written consent to publish potentially identifying information, such as details or the case and photographs, was obtained from the patient(s) or their legal guardian(s).

## Funding

Funding was received for this work.

## Intellectual property

We confirm that we have given due consideration to the protection of intellectual property associated with this work and that there are no impediments to publication, including the timing of publication, with respect to intellectual property. In so doing we confirm that we have followed the regulations of our institutions concerning intellectual property.

## Conflicts of interest

No conflict of interest exists.

## Declaration of competing interest

All authors have no financial disclosures.
